# Evaluation of Multidrug-Resistant *P. aeruginosa* in Healthcare Facility Water Systems

**DOI:** 10.3390/antibiotics10121500

**Published:** 2021-12-07

**Authors:** Maria Luisa Cristina, Marina Sartini, Elisa Schinca, Gianluca Ottria, Beatrice Casini, Anna Maria Spagnolo

**Affiliations:** 1 Department of Health Sciences, University of Genoa, Via Pastore 1, 16132 Genoa, Italy; cristinaml@unige.it (M.L.C.); elisa.schinca@unige.it (E.S.); gianluca.ottria@unige.it (G.O.); am.spagnolo@unige.it (A.M.S.); 2 S.S.D. U.O. Hospital Hygiene, E.O. Ospedali Galliera, 16128 Genova, Italy; 3 Department of Translational Research and New Technologies in Medicine and Surgery, University of Pisa, 56126 Pisa, Italy; beatrice.casini@unipi.it

**Keywords:** *P. aeruginosa*, multidrug resistance, healthcare water system

## Abstract

According to the WHO, *P. aeruginosa* is one of the antibiotic-resistant bacteria that represent the biggest threat to public health. The aim of the study was to establish the prevalence of antibiotic-resistant *P. aeruginosa* in the water systems of various healthcare facilities over the course of nine years. A total of 4500 tap water system samples were taken from seventeen healthcare facilities. The culture method was used to detect *P. aeruginosa*, and the isolates were then tested for antibiotic resistance using the standardised disc diffusion method. Eleven antibiotics from five different classes were tested. *P. aeruginosa* was found to have contaminated 2.07% (no. 93) of the water samples. The majority of positive samples came from the dental units (30.11%) and the ward kitchens (23.66%). Considering the total isolates, 56.99% (no. 3) were resistant to at least one of the antibiotics tested. A total of 71.43% of *P. aeruginosa* isolated from water emerging from dental unit handpieces was antibiotic-resistant, with 45% of it resistant to ≥3 classes of antibiotics. Out of the total isolates, 19.35% showed resistance to carbapenems. It would be advisable to systematically screen tap water for opportunistic micro-organisms such as *P. aeruginosa*, as many countries already do, including this in the Water Safety Plan.

## 1. Introduction

Healthcare-acquired infections (HAIs), particularly in the critical care setting, have become increasingly common over recent decades, with Gram-negative bacterial infections presenting the highest incidence among them [1]. *P. aeruginosa* is one of the most frequent and serious causes of HAIs (e.g., respiratory and urinary tract, skin and soft tissues, ear and eye infections, and bacteriemia), which particularly affect immunocompromised patients [2,3,4].

At-risk patients include neonates, patients with deep neutropenia, severely burned patients, patients with invasive devices (e.g., vascular and urinary catheters, endotracheal tubes, ventilators), and patients who have underlying pulmonary disease such as bronchiectasis and cystic fibrosis [5].

The emerging presence of *P. aeruginosa* multi-drug-resistant (MDR) isolates, resistant to almost all antimicrobials used for hospital patients, has attracted the attention of many researchers in recent decades [6]. *P. aeruginosa* presents multiple resistance mechanisms, either intrinsic or acquired, frequently with high resistance rates affecting several classes of antibiotics, including carbapenems [7,8].

Various hospital-acquired infection outbreaks due to *P. aeruginosa* have been reported [9,10,11,12,13]. Different potential environmental reservoirs of this micro-organism have been described in healthcare settings (aerosols, taps, basin and shower drains, respiratory equipment, humidifiers, endoscopes and endoscope washers, hydrotherapy pools, dental units, etc.) [11,14,15,16,17].

In greater detail, water sources and water-related devices are often contaminated with pathogens [18,19,20], which may be responsible for healthcare-associated infections, including *P. aeruginosa* [21,22,23,24,25,26,27].

It has been estimated that 20% of nosocomial pneumonias are caused by waterborne *P. aeruginosa* in the US, resulting in a conservative annual mortality of approximately 1400 individuals [28]. Ambrogi et al. [22]. reported a cluster of five cases of infection by *P. aeruginosa* expressing VIM carbapenemases (VIM-PA) in a nephrology intensive care unit transmitted via hands and associated with contaminated tap water.

Drinking water quality is subject to numerous regulations based on its lifetime health effects in the general population. However, with regard to people with increased susceptibility to infection, insufficiently broad water quality indicators are used (e.g., they do not include opportunistic pathogens), and there is a lack of guidelines covering all healthcare settings [18,21]. Only a small number of European countries (United Kingdom, France) and the US Centers for Disease Control and Prevention have drawn up guidelines for water quality in healthcare facilities [29,30,31]. In Germany and France, environmental surveillance of water systems is an integral part of their infection control programmes. For *P. aeruginosa* specifically, the target value is <1 CFU/100 mL [30]. In Italy, there is no specific legislation regarding the control of water in healthcare facilities. Only a small number of studies have been carried out to date relating to the environmental surveillance of antibiotic-resistant *P. aeruginosa* in healthcare facilities, even limited to a specific type of environmental reservoir. The aim of this study was to investigate the prevalence and antimicrobial susceptibility profiles of *P. aeruginosa* isolates obtained from water samples collected from different healthcare facilities in the Liguria region, Northern Italy, over a nine-year period. Since it was not the aim of the study, we did not carry out a clinical surveillance of *P. aeruginosa* infections during the monitoring period.

## 2. Results

Out of the 4500 water samples taken in various healthcare facilities, 2.07% (no. 93) were contaminated with *P. aeruginosa*. Specifically, only 0.40% of inlet water samples were found to be contaminated with *P. aeruginosa*.

Of the isolates, sixty-eight came from the cold water circuit, while the remaining twenty-five were from the hot water circuit. Among the various sampling sites, the highest percentage of positivity (16%) was found in dental units. None of the water samples from taps fitted with absolute filters, such as those in the operating theatres and intensive care units/resuscitation units, showed microbial contamination.

The majority of *P. aeruginosa* isolates came from the dental units (30.11%) and the water system in the ward kitchens (23.66%).

Considering the total isolates, 56.99% (no. 53) were resistant to at least one of the antibiotics tested; of these, the highest percentages of antibiotic-resistant isolates (37.74% and 28.30%) came from dental units and ward kitchens, respectively (Table 1).

The difference between the number of antibiotic-resistant isolates in the dental units and those found at the other sampling points was borderline significant (Pearson chi2 = 3.4076; *p* = 0.052).

Water isolates from the neonatology ward, rehabilitation pool, and patient toilets were susceptible to all antibiotics tested.

**Figure 1:**
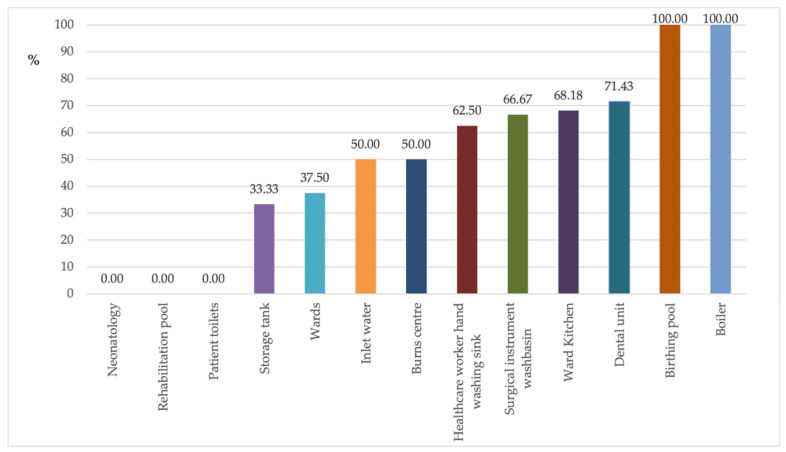
Percentage of antibiotic-resistant *P. aeruginosa* in the individual sampling sites.

Figure 1 shows the percentage of isolates for each sampling site. In the dental units (no. 28) and ward kitchens (no. 22), 71.43% and 68.18% of isolates, respectively, showed antibiotic resistance.

**Figure 2:**
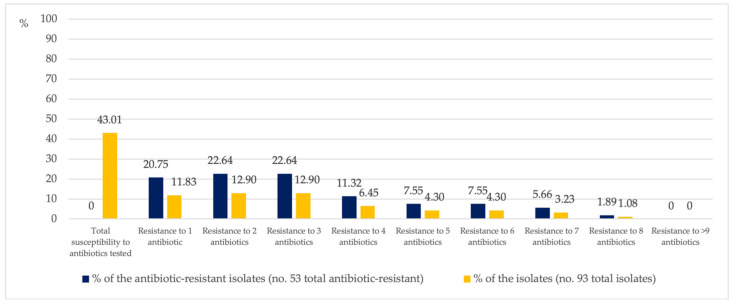
Percentage of *P. aeruginosa* isolates simultaneously resistant to between one and eight antibiotics.

Of the resistant isolates, only 20.75% were resistant to one antibiotic and 22.64% were resistant to two or three antibiotics. A decreasing percentage of isolates showed resistance to more than three antibiotics. In total, 1.89% of the resistant isolates were resistant to eight of the eleven antibiotics tested (Figure 2) and came from the water circuit of a hospital ward kitchen.

The percentage of antibiotic resistant strains has changed over the years, but in a discontinuous way (Figure S1, Supplementary Materials). However, based on the observed data, the probability of occurrence of resistant strains increases by 1.2 over the years (OR = 1.20 [95% CI, 1.01–1.43], *p* = 0.0351).

Table 2 shows the antibiotic-resistant characteristics relative to the *P. aeruginosa* isolates.

Considering the total number of antibiotic-resistant isolates, Aminoglycosides are the class of antibiotics with the lowest percentage of resistance, with no resistance at all being observed for Amikacin. On the other hand, the highest percentages of resistance were recorded for the Penicillins: Piperacillin (75.47%) and Piperacillin/Tazobactam (69.81%).

Furthermore, almost half of the resistant isolates showed resistance to Cefepime (Cephalosporins) and Levofloxacin (Fluoroquinolones).

### 2.1. Multiresistant P. aeruginosa

Out of the total isolates, 17 (18.28%) were multiresistant, being resistant to ≥3 groups of antibiotics at the same time (Table 3, grey area).

Table 4 shows the antibiotic-resistance profile of the *P. aeruginosa* isolate and the multiple antimicrobial resistance (MAR) index. *P. aeruginosa* exhibited twenty-five antibiotic-resistant patterns with the MAR index ranging from 0.09 to 0.73; thirty-one isolates (58.49%) fell into the MAR index >0.2 category.

At the most critical sites in terms of healthcare impact and/or possibility of persistence due to easier biofilm formation, several multiresistant isolates were found. In the water coming from the hand shower in a hospital burns unit, *P. aeruginosa* was resistant to four classes of antibiotics (seven out of eleven antibiotics tested).

**Figure 3:**
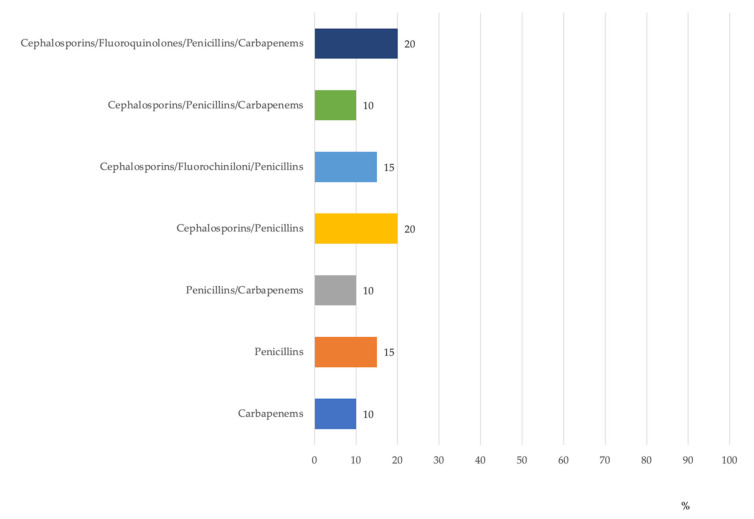
Percentage distribution of antibiotic-resistant *P. aeruginosa* isolates in dental units by antibiotic groups.

Another critical control point was the healthcare workers’ hand-washing sink, where 20% of resistant isolates (no. = 5) were multidrug-resistant (resistant to four classes of antibiotics). Out of the antibiotic-resistant isolates in the water within the internal circuit of dental units, 45% were resistant to ≥3 classes of antibiotics (Figure 3).

### 2.2. Carbapenem Resistance

**Figure 4:**
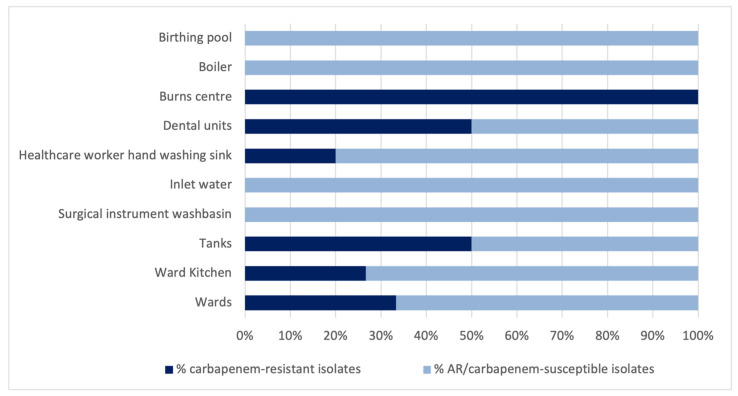
Percentage distribution of carbapenem-resistant *P. aeruginosa* isolates in the various sampling points.

19.35% of the total *P. aeruginosa* isolates were found to be resistant to carbapenems. Among the antibiotic-resistant isolates (no. 53), carbapenem resistance was 33.96%. Regarding the latter, Figure 4 shows the percentage of carbapenem-resistant isolates (R) and of antibiotic-resistant but carbapenem-susceptible isolates (AR) on the basis of the sampling point.

In the dental units, ten P. aeruginosa isolates showed resistance to carbapenems. The difference between the number of carbapenem-resistant isolates in the dental units and those found at the other sampling points was borderline significant (Pearson chi2 = 3.6837; *p* = 0.053).

## 3. Discussion

Antimicrobial resistance is now one of the most important public health problems in both human and veterinary medicine and is set to become one of the major health challenges of the coming decades.

To tackle the problem of antibiotic resistance, the WHO has promoted the “One Health” strategy, integrating all relevant sectors, from human and veterinary use to food, agricultural and environmental safety. In Italy, where the phenomenon of antibiotic resistance is among the highest in Europe, at levels almost always above average, the Ministry of Health has published the “National Plan to Combat Antimicrobial Resistance (PNCAR) 2017–2020” [32] which addresses the problem from a general, human, and veterinary medicine perspective.

The spread of multidrug-resistant *P. aeruginosa* is particularly concerning. Over recent years, the worldwide spread of so-called “high-risk clones” of multidrug-resistant or extensively drug-resistant (MDR/XDR) *P. aeruginosa* has become a public health threat that needs to be urgently and decisively studied and managed [3].

According to the WHO [7], *Pseudomonas aeruginosa* is one of the antibiotic-resistant bacteria that represents the biggest threat to public health. It is intrinsically resistant to most antimicrobial agents due to its selective ability to prevent various antibiotic molecules from penetrating its outer membrane or extruding them if they enter the cell. It also exhibits acquired resistance mechanisms.

The ECDC’s European Antimicrobial Resistance Surveillance Network (EARS-Net) reported that, for 2019, 31.8% of *P. aeruginosa* isolates in the EU/EEA were resistant to at least one of the antimicrobial groups under regular surveillance (piperacillin + tazobactam, fluoroquinolones, ceftazidime, aminoglycosides, and carbapenems). The highest EU/EEA population-weighted mean resistance percentage in 2019 was reported for fluoroquinolones (18.9%), followed by piperacillin + tazobactam (16.9%), carbapenems (16.5%), ceftazidime (14.3%), and aminoglycosides (11.5%) [8].

ECDC data from 2018 [33], which is more detailed for individual countries in the EU/EEA, showed *P. aeruginosa* combined resistance (resistance to three or more antimicrobial groups among piperacillin ± tazobactam, ceftazidime, fluoroquinolones, aminoglycosides and carbapenems) in the EU/EEA of 12.8%, with considerable variation between countries, from 0% in Iceland to 49.4% in Romania. In Italy, 14.9% of 2006 *P. aeruginosa* clinical isolates were multiresistant.

Because *P. aeruginosa* is one of the main agents of nosocomial infections and is increasingly resistant to antibiotics, environmental reservoirs in hospital settings are of great concern.

To date, some studies have been conducted on the circulation and/or the prevalence of multidrug-resistant *P. aeruginosa* in healthcare facility environmental reservoirs (e.g., water) [24,25,26], but they are still few in number.

Our study has revealed the presence of *P. aeruginosa* in different healthcare water samples (2.07%), including resistant strains. The percentage of positivity for *P. aeruginosa* found in this study is within the range of prevalence observed in similar investigations [18,25,26].

Out of the *P. aeruginosa* isolates analysed, 56.99% (no. = 53) were resistant to at least one of the antibiotics tested, of which 37.74% and 28.30% were found in dental units and ward kitchens, respectively.

*P. aeruginosa* exhibited twenty-five antibiotic-resistant patterns with the MAR index ranging from 0.09 to 0.73. Thirty-one isolates (58.49%) fell into the MAR index >0.2 category. The MAR index is a good tool for health risk assessment which identifies whether isolates are from a region of high or low antibiotic use. A MAR index >0.2 indicates a “high-risk” source of contamination [34,35].

Out of the total isolates, 18.28% were multiresistant, being resistant to ≥3 groups of antibiotics. In the most critical sites in terms of healthcare impact and/or possibility of persistence due to biofilm formation, several multiresistant isolates were found. Twenty percent of *P. aeruginosa* isolates from healthcare workers’ handwashing tap water were resistant to four classes of antibiotics.

In water from the hand shower of a hospital burns unit, *P. aeruginosa* was resistant to seven out of eleven antibiotics tested, belonging to four distinct classes. Infections caused by MDR bacteria act as a risk factor for mortality in burns patients. Out of all MDR bacteria, *P. aeruginosa* proved the most significant because this bacterium showed the most growth on the moist surface of burn wounds and is highly pathogenic in immunocompromised patients [36].

Considering the other critical control points monitored in our study, it emerged that 71.43% of *P. aeruginosa* isolated from water flowing from dental unit handpieces was antibiotic-resistant, and 45% of this was resistant to ≥3 classes of antibiotics.

An important characteristic of *P. aeruginosa* is its ability to form biofilms as an adaptation to adverse environmental conditions [37,38,39]. In dental units, the water conduit can be composed of approximately 6 m of narrow-bore flexible polyurethane or PVC plastic tubing (1/16 in. or 2 mm diameter), which encourages biofilm formation of a wide variety of microorganisms [39,40,41], including antibiotic-resistant *P. aeruginosa*. This microorganism can be responsible for infections in immunocompromised patients treated at dental units harbouring these organisms [42].

The circulation of carbapenem-resistant strains of *P. aeruginosa* is particularly concerning as infections sustained by these strains are difficult to treat, both because there are often no adequately effective and safe therapeutic options available and because the associated mortality rate is higher than for infections with carbapenem-sensitive *P. aeruginosa* [43].

In Italy, the percentage of invasive isolates with resistance to carbapenems (imipenem or/and meropenem) was between 10% and <25% in 2019 [8].

In our study, 19.35% of the total *P. aeruginosa* water isolates were found to be resistant to carbapenems. Among the antibiotic-resistant isolates (no. 53), carbapenem resistance was 33.96%.

The data collected in this study highlight the importance of environmental surveillance of antibiotic-resistant and MDR microorganisms, together with the adoption of measures to prevent environmental contamination.

According to WHO guidelines [7], measures to prevent the transmission of multiresistant *P. aeruginosa* in healthcare facilities should include at least the following: hand hygiene (with the appropriate use of alcohol-based solutions), contact precautions, patient isolation (single room or cohort), environmental cleanliness, and surveillance.

In order to specifically mitigate the risks of water contamination by microorganisms responsible for care-related infections, the implementation of a Water Safety Plan (WSP) is essential. The main elements of this plan should comprise active infection surveillance, the adoption of disinfection procedures or other water treatments, the maintenance of water networks, and the scheduling of periodic checks of the water withdrawn at the most significant points of the tap water system [44,45].

In light of the findings that emerged from this study and international scientific evidence, it would be advisable to systematically screen tap water for opportunistic micro-organisms such as *P*. *aeruginosa*, as many countries already do, including this in the Water Safety Plan.

Understanding potential environmental reservoirs of infectious bacterial species and the role that water and water-related devices play as reservoirs for antimicrobial-resistant bacteria is crucial to prevent HAIs.

A limitation of our study was the possible underestimation of microbial contamination of water leaving taps fitted with POU filters (e.g., in the operating theatre and intensive care/resuscitation unit) upstream from the taps, as sampling in these cases was carried out without removing the filter.

Various authors have highlighted how prolonged use of point-of-use filters may create a water flow slowdown and retrograde contamination [46]. Therefore, sampling the water after the removal of the absolute filter and allowing the water to flow would have led to possible environmental dispersion of potentially pathogenic microorganisms concentrated in the end of the taps, constituting a possible health risk for severely debilitated patients.

Another limitation of the study was the lack of surveillance of cases of nosocomial *P. aeruginosa* infection in the healthcare facilities examined during the nine years of monitoring. The use of molecular biology on clinical and environmental isolates could have made it possible to highlight clonal relationships between patient and tap water isolates and thus the potential water-based origin of the infections. On the other hand, the opposite route of transmission cannot be excluded. Some scientific evidence has shown that transmissions of *P. aeruginosa* can occur both from tap to patient and from patient to tap [47].

## 4. Materials and Methods

### 4.1. Setting

Between January 2011 and January 2020, a total of 4500 water samples were collected from seventeen healthcare facilities in Northern Italy (region of Liguria): eight hospitals, three nursing homes, and six outpatient clinics.

Various points of the healthcare facility water systems were sampled: inlet water, storage tanks, birthing pools, boilers, rehabilitation pools, and various critical points such as medical wards, neonatology, operating theatres (surgical instrument washing sink, surgical scrub sink), intensive care/resuscitation, hand washing for health workers, burn centre, dental units, ward kitchens, patient toilets, etc.

Water sampling was carried out as part of the routine surveillance plan of water quality in health care facilities. The sampling was conducted every six months (in spring/summer and in autumn/winter) both on the cold and hot water circuits for a total of about 500 samples per year. The hot water circuit was equipped with chlorination systems within the healthcare facilities, while the cold water circuit did not undergo any additional disinfection with respect to disinfection already applied within the water supply system.

### 4.2. Water Sampling and Microbiological Analysis

The water was drawn from taps, showers or by immersion (in the case of storage tanks). In the case of taps, the sampling was carried out after removing the diffuser head (when present), flushing the tap, and letting the water run for 1–3 min.

In taps equipped with absolute filters, water was taken without dismantling them and therefore downstream of them.

In the case of dental units, the water was taken from handpieces. From each sampling point, water was collected in sterile disposable plastic (polyethylene) bottles containing sodium thiosulphate to inhibit the action of residual chlorine in the sampled water. The samples were transported in heat-insulated containers under refrigerated conditions and analysed within two hours of their arrival at the laboratory.

The samples were analysed for the detection of *Pseudomonas aeruginosa* using a standard method based on the membrane filtration technique (UNI EN ISO 16266) [48]. Briefly, the water sample (100 mL) was filtered through a cellulose ester membrane (0.45 μm porosity, 47 mm diameter); the membrane was then placed on Pseudomonas CN agar medium (Liofilchem, Roseto degli Abruzzi (TE), Italy), which is a selective medium for *P. aeruginosa*, and subsequently cultured at 36 ± 2 °C for 44 ± 4 h before colony counting.

Blue/green pyocyanin-producing colonies were counted as confirmed *P. aeruginosa*. Fluorescent non-pyocyanin-producing or reddish-brown colonies were recorded as presumptive *P. aeruginosa* and subjected to confirmation tests according to ISO 16266.

### 4.3. Antibiotic Susceptibility Testing

The *P. aeruginosa* isolates were gradually frozen at −80 °C as they were collected, and then simultaneously revitalised and tested for antibiotic resistance in 2020.

The *P. aeruginosa* isolates were tested using the European Committee on Antimicrobial Susceptibility Testing (EUCAST) standardised disc diffusion method.

These tests were performed on Mueller−Hinton agar using the Kirby−Bauer disc diffusion technique. All plates were incubated at 35 °C ± 1 °C for 16 ± 18 h. Zones of inhibition around the disk were measured and interpreted as proposed by the latest EUCAST breakpoint criteria.

Eleven antibiotics from five different classes were tested: Amikacin 30 µg, Gentamicin 10 µg, Tobramycin 10 µg, Cefepime 30 µg, Ceftazidime 10 µg, Ciprofloxacin 5 µg, Levofloxacin 5 µg, Piperacillin 30 µg, Piperacillin/Tazobactam (36 µg), Imipenem 10 µg, Meropenem 10 µg.

*Pseudomonas aeruginosa* ATTC 27853 was used for quality control.

The Multiple Antimicrobial Resistance (MAR) index has been calculated as the ratio between the number of antibiotics that an isolate is resistant to and the total number of antibiotics the organism is exposed to.

### 4.4. Statistical Analysis

The data were processed using the statistical programme STATA SE14^TM^ (StataCorp, College Station, Texas, USA). Descriptive statistics were performed. The collected information was summarised using frequency and percentage for qualitative data. Differences between antibiotics resistance isolates were evaluated by means of the non-parametric chi-square test and Fisher exact test. A *p* value less than 0.05 was considered statistically significant.

## Figures and Tables

**Table 1:** *P. aeruginosa* isolates in the various water sampling points.

Sampling Points	% of *P. aeruginosa* Isolates Tested (No.)	% of Antibiotic-Resistant *P. aeruginosa* Strains (No.)
Dental unit	30.11 (28)	37.74 (20)
Ward kitchen	23.66 (22)	28.30 (15)
Ward	8.60 (8)	5.66 (3)
Healthcare worker hand-washing sink	8.60 (8)	9.43 (5)
Storage tank	6.45 (6)	3.77 (2)
Neonatology	4.30 (4)	0
Rehabilitation pool	4.30 (4)	0
Surgical instrument washing sink	3.23 (3)	3.77 (2)
Birthing pool	3.23 (3)	5.66 (3)
Inlet water	2.15 (2)	1.89 (1)
Burns centre	2.15 (2)	1.89 (1)
Patient toilets	2.15 (2)	0
Boiler	1.08 (1)	1.89 (1)
Total	100 (93)	100 (53)

**Table 2:** *P. aeruginosa* isolates resistant (R) to the various antibiotics tested.

Antibiotic Group	Antibiotics Tested	No. Resistant Isolates	% of the Antibiotic-Resistant Isolates (No. 53 Total Antibiotic-Resistant)	% of the Isolates (No. 93 Total Isolates)
Aminoglycosides	Amikacin	0	0	0
	Gentamicin	1	1.89	1.08
	Tobramycin	1	1.89	1.08
Cephalosporins	Cefepime	25	47.17	26.88
	Ceftazidime	11	20.75	11.83
Fluoroquinolones	Ciprofloxacin	7	13.21	7.53
	Levofloxacin	24	45.28	25.81
Penicillins	Piperacillin	40	75.47	43.01
	Piperacillin/Tazobactam	37	69.81	39.78
Carbapenems	Imipenem	18	33.96	19.35
	Meropenem	4	7.55	4.30

**Table 3:** Percentage of *P. aeruginosa* isolates resistant to 1, 2, 3, 4, 5 groups of antibiotics tested.

Resistance Pattern	No. Isolates	% of the Antibiotic-Resistant Isolates (No. 53 Total Antibiotic-Resistant)	% of the Isolates (No. 93 Total Isolates)
Total susceptibility to the antibiotics tested	40	-	43.01
Resistance to 1 group of antibiotics	20	37.74	21.51
Resistance to 2 groups of antibiotics	16	30.19	17.20
Resistance to 3 groups of antibiotics	8	15.09	8.60
Resistance to 4 groups of antibiotics	9	16.98	9.68
Resistance to 5 groups of antibiotics	0	0	0
Total	93		

**Table 4:** *P. aeruginosa* isolate antibiotic-resistance profiles (no. 53) and MAR index.

Pattern	No. of Isolates Showing Profile	Resistance Phenotype	MAR Index
1	4	PIP	0.09
2	5	LVX	0.09
3	1	IPM	0.09
4	1	TZP	0.09
5	1	CIP, LVX	0.18
6	7	PIP, TZP	0.18
7	1	TOB, CPM	0.18
8	1	LVX, TZP	0.18
9	1	IPM, MEM	0.18
10	3	PIP, TZP, IPM	**0.27**
11	1	LVX, PIP, TZP	**0.27**
12	6	CPM, PIP, TZP	**0.27**
13	2	CPM, LVX, PIP	**0.27**
14	1	GEN, CIP, LVX	**0.27**
15	1	LVX, PIP, TZP, IPM	**0.36**
16	2	CPM, CAZ, PIP, TZP	**0.36**
17	2	CPM, PIP, TZP, IPM	**0.36**
18	1	PIP, TZP, IPM, MEM	**0.36**
19	1	CPM, CIP, LVX, PIP, TZP	**0.45**
20	2	CPM, CAZ, LVX, PIP, TZP	**0.45**
21	1	CPM, LVX, PIP, TZP, IPM	**0.45**
22	4	CPM, CAZ, LVX, PIP, TZP, IPM	**0.55**
23	2	CPM, CAZ, CIP, LVX, PIP, TZP, IPM	**0.64**
24	1	CPM, CIP, LVX, PIP, TZP, IPM, MEM	**0.64**
25	1	CPM, CAZ, CIP, LVX, PIP, TZP, IPM, MEM	**0.73**

AMK: Amikacin 30 µg; CAZ: Ceftazidime 10 µg; CIP: Ciprofloxacin 5 µg; CPM: Cefepime 30 µg; GEN: Gentamicin 10 µg; IPM: Imipenem 10 µg; LVX: Levofloxacin 5 µg; MEM: Meropenem 10 µg; PIP: Piperacillin 30 µg; TOB: Tobramycin 10 µg; TZP: Piperacillin/Tazobactam (36 µg).

## Data Availability

The data presented in this study are available on motivated request from the corresponding author.

## Supplementary Materials

The following are available online at https://www.mdpi.com/article/10.3390/antibiotics10121500/s1, Figure S1: *P. aeruginosa* antibiotic resistant isolates and total of *P. aeruginosa* isolates in the years of observation.
