# Supplementary material for: Evaluation of Multidrug-Resistant P. aeruginosa in Healthcare Facility Water Systems
Source: Antibiotics (Basel). 2021 Dec 7;10(12):1500. doi: 10.3390/antibiotics10121500 (PMC8698422; doi:10.3390/antibiotics10121500)

Figure S1. *P. aeruginosa* antibiotic resistant isolates and total of *P. aeruginosa* isolates in the years of observation.

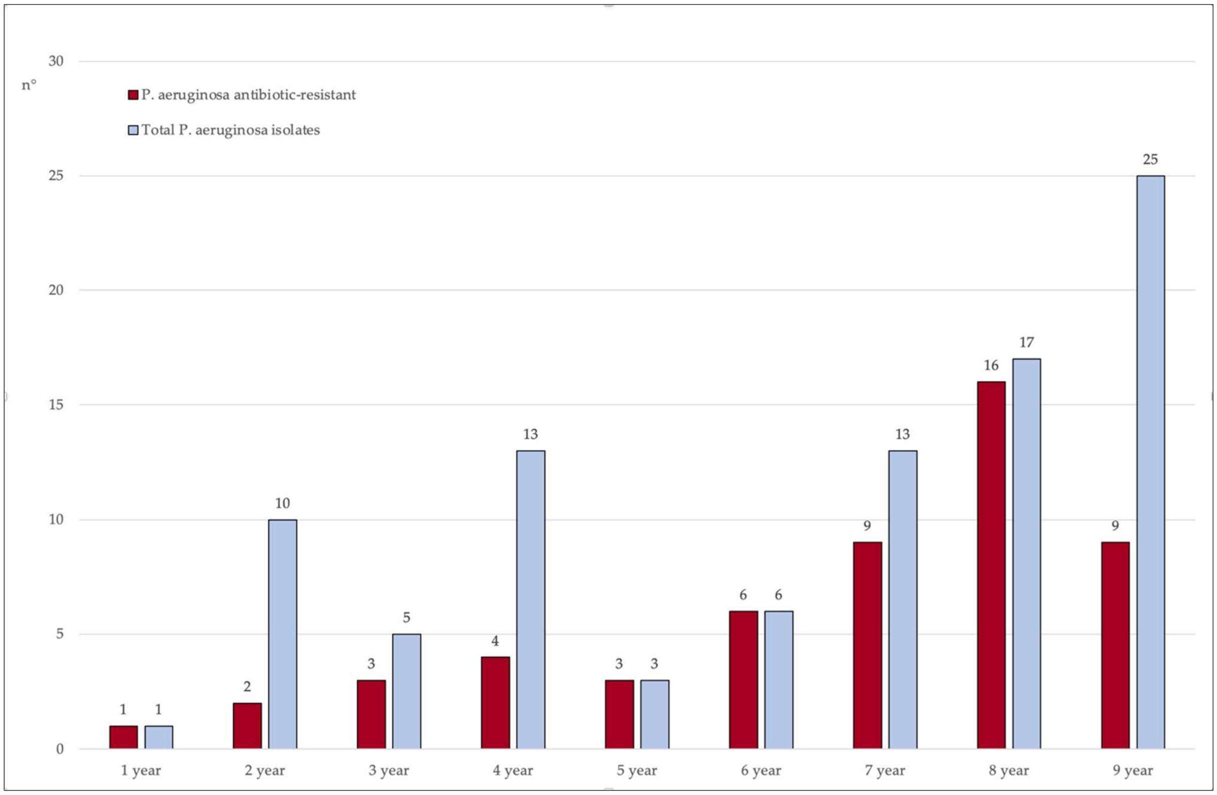

Supplement: Supplementary file 1 [file antibiotics-10-01500-s001.zip › antibiotics-1447227-supplementary.pdf]
